# Hepatic Rupture Induced by Spontaneous Intrahepatic Hematoma

**DOI:** 10.1155/2018/2026846

**Published:** 2018-01-28

**Authors:** Jin-bao Zhou, Wei-bo Chen, Feng Zhu

**Affiliations:** Department of Hepatobiliary Surgery, The Third Affiliated Hospital of Soochow University, Changzhou, Jiangsu 213000, China

## Abstract

The etiology of hepatic rupture is usually secondary to trauma, and hepatic rupture induced by spontaneous intrahepatic hematoma is clinically rare. We describe here a 61-year-old female patient who was transferred to our hospital with hepatic rupture induced by spontaneous intrahepatic hematoma. The patient had no history of trauma and had a history of systemic lupus erythematosus for five years, taking a daily dose of 5 mg prednisone for treatment. The patients experienced durative blunt acute right upper abdominal pain one day after satiation, which aggravated in two hours, accompanied by dizziness and sweating. Preoperative diagnosis was rupture of the liver mass. Laparotomy revealed 2500 mL fluid consisting of a mixture of blood and clot in the peritoneal cavity. A 3.5 cm × 2.5 cm rupture was discovered on the hepatic caudate lobe near the vena cava with active arterial bleeding, and a 5  × 6 cm hematoma was reached on the right posterior lobe of the liver. Abdominal computed tomography (CT) and laparotomy revealed spontaneous rupture of intrahepatic hematoma with hemorrhagic shock. The patient was successfully managed by suturing the rupture of the hepatic caudate lobe and clearing part of the hematoma. The postoperative course was uneventful, and the patient was discharged after two weeks of hospitalization.

## 1. Introduction

Large intrahepatic hematomas are rare, representing 0.2% of all documented hematomas. However, hematomas with a fatal outcome which require urgent management are less [1]. Spontaneous rupture of the intrahepatic hematoma is reported to be a rare but fatal complication; the intrahepatic hematomas or rupture appears in 1 of every 100,000 pregnancies [2], and it is even rarer in adults. Herein, we report a hepatic rupture patient who presented with a nontraumatic intrahepatic hematoma. The patient had a history of systemic lupus erythematosus for five years, taking a daily dose of 5 mg prednisone for treatment. This case indicates that hepatic rupture induced by spontaneous rupture of the intrahepatic hematoma can have a good prognosis by suturing the rupture and removing some hematomas. In addition, long-term use of hormones may be the cause of spontaneous rupture of the intrahepatic hematoma.

## 2. Case Report

**Figure 1:**
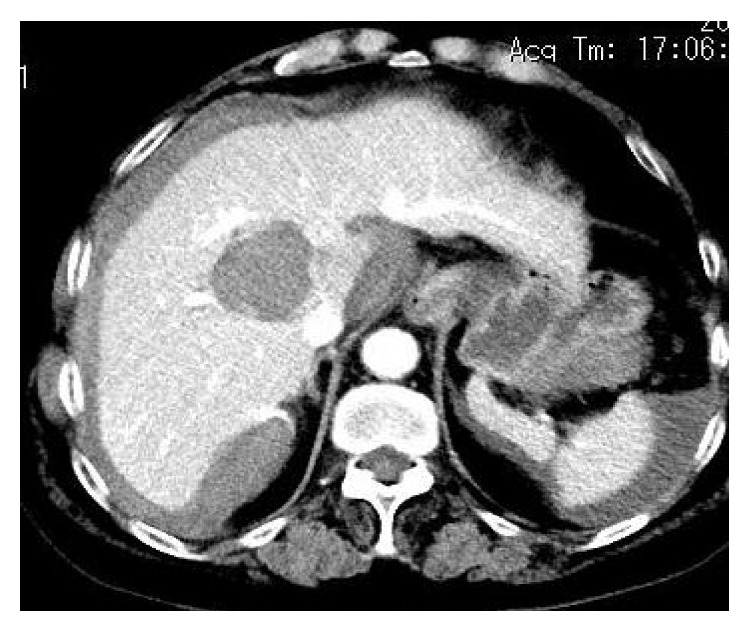
Abdominal CT revealed rupture of the posterior lobe lesion, hemorrhage around the liver, and accumulation of fluid in the abdominal cavity and pelvic cavity.

A 61-year-old female patient experienced acute right upper abdominal durative blunt pain one day after satiation, which aggravated in two hours, accompanied by dizziness and sweating. She was immediately admitted to our hospital. The patient had a heart rate of 101 per minute and a blood pressure of 75/58 mmHg. Abdominal CT revealed rupture of a lesion situated in the hepatic posterior lobe, hemorrhage around the liver, and accumulation of fluid in the abdominal cavity and pelvic cavity (Figure 1). CT scan did not show significant abnormality in the liver three months before admission. The patient had no history of trauma and had a history of systemic lupus erythematosus for five years, taking a daily dose of 5 mg prednisone for treatment. She also had a history of chronic bronchitis and emphysema for many years, which were properly managed under medical control. On physical examination, no pallor or jaundice was noted. Total abdominal tenderness, mild rebound pain, liver and kidney area percussion pain (+), shifting dullness (+), and weak bowel sounds were noted. Examination of the heart and lungs was normal. Laboratory results showed 13.6 g/dl of hemoglobin, 9,510/*μ*l of white blood cell count, and 65,000/*μ*l of platelet count. ALT and AST were 119/*μ*l and 357/*μ*l, respectively. BUN, creatinine, and coagulation function were normal. Preoperative diagnosis was rupture of the liver mass, systemic lupus erythematosus, chronic bronchitis, and emphysema.

**Figure 2:**
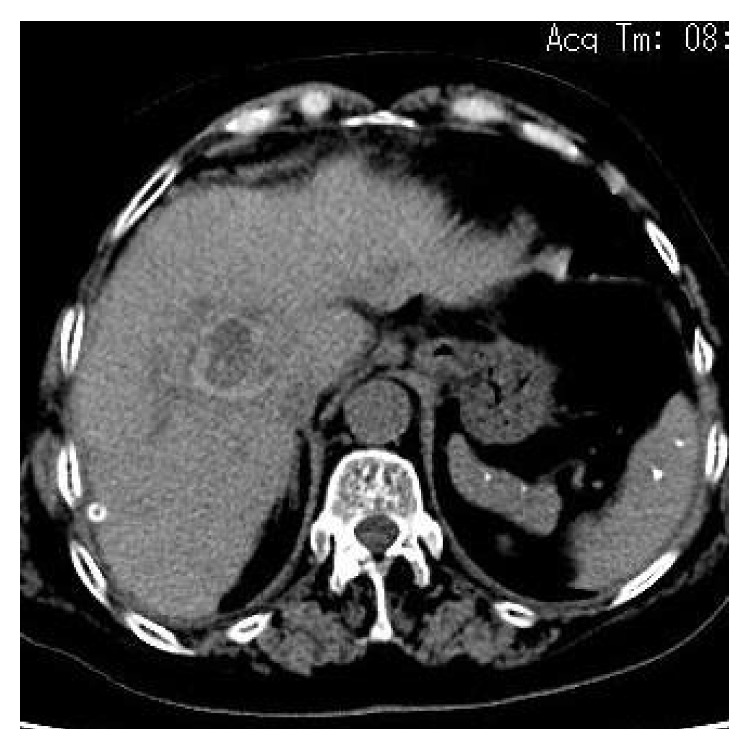
Two weeks after surgery, CT showed obvious absorption of intrahepatic hemorrhage, which was significantly improved compared with previous films.

Fluid and norepinephrine were administrated to raise the blood pressure. The patient was immediately sent to the operating room. Laparotomy revealed 2500 mL fluid consisting of a mixture of blood and clot in the peritoneal cavity. The gallbladder, spleen, stomach, duodenum, small intestine, colon, and pancreas appeared normal. A 3.5 cm × 2.5 cm rupture was discovered on the hepatic caudate lobe near the vena cava with active arterial bleeding, and a 5 cm × 6 cm hematoma was reached on the right posterior lobe of the liver. Through intraoperative exploration, the blood point was considered as the branch of the right posterior hepatic artery. Combined with the patient's clinical signs, preoperative examination, and intraoperative exploration, we excluded hepatocellular carcinoma, hepatic adenoma, and so on as the cause of intrahepatic hematoma, and we believe that intrahepatic hematoma was caused by intrahepatic artery rupture and then spontaneous rupture of the intrahepatic hematoma leading to hepatic rupture. According to the principle of damage control, we carried out conservative treatment by ligating the bleeding artery and suturing the rupture of the hepatic caudate. The postoperative course was uneventful, and the patient was discharged after two weeks of hospitalization. Two weeks after surgery, CT showed obvious absorption of intrahepatic hemorrhage, which was significantly improved compared with previous films (Figure 2).

## 3. Discussion

Spontaneous rupture of the intrahepatic hematoma is a rare but life-threatening complication of a number of disease states [3]. A mechanical injury of an intrahepatic artery branch might be the reason for this unusually severe hemorrhagic complication, and the existing hypertension may aggravate the situation [4]. Intrahepatic hemorrhage has also been documented in the literature as an uncommon complication of hepatic amyloidosis, with the majority of cases occurring after liver biopsy [5]. Spontaneous microhemorrhages within the liver subsequently lead to a final common pathway of capsular distension, hematoma formation, and possible spontaneous rupture [6]. However, the pathogenesis of the intrahepatic hematoma and subsequent rupture is unclear. Anabolic steroids are known to have a profound impact on the liver, including peliosis hepatis, cholestasis, hepatic adenomas, and intrahepatic hematomas [7, 8]. However, there are few reports of the hepatic hematoma induced by anabolic steroids. The New England Journal of Medicine has reported a case of a large hepatic hematoma and subsequent intra-abdominal hemorrhage associated with the abuse of anabolic steroids [9]. In our case, long-term use of prednisone was speculated to be the initiating event. The possible mechanism is that anabolic steroids promote the retention of water and sodium to form hypertension and lead to atherosclerosis, which are the risk factors for inducing intrahepatic arterial rupture to form hematoma, and eventually, hematoma rupture leads to hepatic rupture.

The clinical presentation of the intrahepatic hematoma is not characteristic. Most patients present with right upper quadrant pain [10]. However, due to the rarity of this entity and its variable presentation, most cases are missed and diagnosed only at laparotomy. This case highlights that clinical diagnosis can be difficult, as the symptoms and signs are nonspecific. Radiological adjuncts such as ultrasound, CT, or MRI are useful in arriving at the diagnosis. CT scan is particularly useful in an acute setting as they can define the extent and age of the hematoma. During the first 24–72 h, acute hematomas are hyperattenuating on nonenhanced CT scans, decreasing in attenuation thereafter [4]. Once the diagnosis has been made, aggressive early management is crucial. Resuscitation with intravenous fluids and transfusion of blood as well as fresh frozen plasma and platelets may be necessary, especially in patients who present with hemorrhagic shock. Historically, surgical intervention has been the standard of treatment in all cases. Operative interventions include evacuation of hematoma, packing, application of gelfoam- or collagen-impregnated materials, oversewing of lacerations, and drainage to prevent reaccumulation of blood [11].

To the best of our knowledge, the majority of nonruptured spontaneous intrahepatic hematomas are hemodynamically stable, and patients can be cured conservatively [12]. A contained hematoma in a stable patient is treated with fluid and/or blood transfusion [13, 14]. However, a dense intrahepatic hematoma may rupture spontaneously or secondary to trivial trauma during labor or convulsions, leading to catastrophic life-threatening hemorrhage. As we know, surgery is reserved for cases of impending rupture or ruptured hematoma [13]. Once the hematoma ruptures, many patients need surgery to remove the hematoma [15]. As for our case, the patient has a past medical history of long-term use of prednisone, which may be the inducement of spontaneous rupture of the liver hematoma. And the laboratory results showed the patient with good liver function, and we believe that the slight elevation in liver enzymes is due to compression of the liver by the large hematoma rather than the cause of the hematoma. Therefore, we just sutured the rupture of the hepatic caudate lobe and cleared part of the hematoma after the ligation of the bleeding artery. One week after the surgery, the abdominal CT revealed no hematoma. Therefore, we proved that conservative treatment for hepatic rupture induced by spontaneous intrahepatic hematoma was valid, which is meaningful to reduce the pain and the economic and benefits for the patients.

In conclusion, although intrahepatic hematomas are rare, they do have the potential to rupture, which can rapidly lead to hemodynamic compromise and hepatic rupture, or even death. Therefore, a period of close observation is required in the acute setting. A thorough diagnostic workup, with CT and/or MRI, will guide surgeons to an appropriate treatment strategy. Sometimes, conservative treatment such as suturing the rupture and clearing part of the hematoma in a simple way is preferred in therapy options.
